# Culture collection containing 1,240 isolates from healthy Canadian laying hens

**DOI:** 10.1128/mra.01203-24

**Published:** 2024-12-27

**Authors:** Zhixuan (Tiffany) Feng, Jinha Suh, Jennifer Ronholm

**Affiliations:** 1 Faculty of Agricultural and Environmental Sciences, Macdonald Campus, McGill University, Montréal, Québec, Canada; Wellesley College Department of Biological Sciences, Wellesley, Massachusetts, USA

**Keywords:** microbiome, laying hen, culture, deep culturing

## Abstract

The pure culture of prokaryotes is essential to understanding their physiology. To facilitate research into better understanding the roles of individual bacteria in the gastric microbiome in chickens, we have established a culture collection of 1,240 isolates from fecal samples collected from healthy laying hens from across Canada.

## ANNOUNCEMENT

Metagenomics has rapidly advanced our understanding of the chicken microbiome; however, pure cultures must be obtained to understand the microbiome in greater depth. Pure cultures are required to assign unidentified sequences to specific organisms, probe relationships between specific bacteria—this is particularly important when it comes to pathogen-microbiome interactions, explore experimental models, and develop novel microbiome-based therapeutic strategies. Therefore, we performed deep culturing on six fecal samples collected from seemingly healthy laying hens using the protocols previously described for deep culturing the human gastric microbiome (1). This culturing effort yielded 1,240 isolates that represent 130 different bacterial and fungal species, 5 of which have not been previously reported to be isolated from the chicken intestinal microbiome. This culture collection will serve as a resource for the laying hen microbiome research community to obtain pure cultures of a diverse array of isolates.

Fecal samples were collected, by egg producers, from six different laying hens by placing the chicken on a clean ethanol sterilized surface and waiting for it to naturally defecate. After defecation, the fecal sample was divided into four aliquots using a sterile spatula, and an aliquot was placed into each of ½ tryptic soy broth (TSB) (BD Company), amies transport medium (Thermo Fisher Scientific), thioglycolate medium with hemin and vitamin K (Anaerobe System), and anaerobic tissue transport medium (Anaerobe System) and shipped to the lab in Montreal, Canada. Upon arrival in the lab, the sample in TSB and amies transport medium were mixed for aerobic culture, and the sample of thioglycolate medium and anaerobic tissue transport medium were combined for anaerobic culture. Samples for aerobic culture were immediately diluted to 10^−3^, 10^−5^, and 10^−7^ in phosphate-buffered saline (PBS), and 200 µL was spread-plated onto each of Brain Heart Infusion Agar (Thermo Fisher Scientific), Brucella Agar (BD Company), Drigalski Lactose Agar (Thermo Fisher Scientific), EMB Agar (BD Company), Glucose Bromcresol Purple Agar (MilliporeSigma), Hektoen Enteric Agar (HiMedia), Legionella CYE Agar (Thermo Fisher Scientific), MacConkey Agar (BD Company), Marine 2216 Agar (BD Company), Modified Letheen Agar (Hardy Diagnostics), Mueller Hinton Agar (BD Company), Orange Serum Agar (Hardy Diagnostics), R2A Agar (BD Company), Regan-Lowe Agar (BD Company), Salmonella Shigella agar (BD Company), Sheep Blood Agar (BD Company), Thiosulfate-citrate-bile-sucrose agar (BD Company), and Yersinia selective agar (Thermo Fisher Scientific). Samples for anaerobic culture were inoculated onto Drigalski Lactose Agar, EMB Agar, Glucose Bromcresol Purple Agar, Hektoen Enteric Agar, MacConkey Agar, Marine Agar, Modified Letheen Agar, Orange Serum Agar, R2A Agar, Schaedler Agar (BD Company), Sheep Blood Agar, Thioglycolate Agar (HiMedia), Thiosulfate-citrate-bile-sucrose agar, Wilkins-Chalgren Anaerobic Agar (Thermo Fisher Scientific), and Yersinia selective agar. All culturing and incubation of anaerobic samples took place in an anaerobic chamber using an atmospheric gas mix containing 85% N_2_, 5% CO_2_, and 5%H_2_ (Linde Canada). Each plate was incubated at 37°C for 48 hrs. Aerobically shipped samples were also used to inoculate two tubes of Plus Aerobic/F Medium (BD Company) with defibrinated sheep blood (Fisher Scientific/CEDARLANE) and sterilized rumen fluid (Fisher Scientific), while anaerobically shipped samples were used to inoculate two tubes of Lytic Anaerobic Medium bottles (BD Company) containing defibrinated sheep blood and sterilized rumen fluid. One tube from each culture condition was heat shocked at 80°C for 1 hr, and then both tubes were incubated at 37°C for 28 days. At days 3, 7, 14, 21, and 28, samples (1 mL) were taken from enrichment cultures and plated onto the media listed above for either aerobic or anaerobic cultures. For the samples from aerobic enrichment culture conditions with pre-treatment, we spread the sample onto Sheep Blood Agar, Brain Heart Infusion Agar, Modified Letheen Agar, and Glucose Bromcresol Purple Agar. For the samples from anaerobic enrichment culture conditions with pre-treatment, we plated samples onto Brain Heart Infusion Agar, Modified Letheen Agar, Wilkins-Chalgren Anaerobic agar, and Glucose Bromcresol Purple Agar.

Each isolated colony was streaked onto the same agar used to isolate it, and pure well-isolated colonies were enriched in TSB for 48 hrs at 37°C, mixed with 50% glycerol, and stored at −80°C. Isolates were identified by MALDI-TOF MS or by 16S rRNA gene sequencing.

Any isolates from this collection (summary in Table 1) will be made available to the requestor at cost by emailing the corresponding author. The contents of the collection can be viewed at https://doi.org/10.5281/zenodo.14289579.

**TABLE 1:** Summary of strain isolate from healthy laying hens

Kingdom	Phylum	Number of species	Number of strains
Fungi	Ascomycota	1	3
Fungi	Basidiomycota	1	1
Bacteria	Actinomycetota	12	19
Bacteria	Bacteroidota	2	2
Bacteria	Bacillota	63	337
Bacteria	Pseudomonadota	51	878

## References

[1] Lagier J-C, Khelaifia S, Alou MT, Ndongo S, Dione N, Hugon P, Caputo A, Cadoret F, Traore SI, Seck EH, et al.. 2016. Culture of previously uncultured members of the human gut microbiota by culturomics. Nat Microbiol 1:16203. doi:10.1038/nmicrobiol.2016.203 27819657 PMC12094094

